# Diagnostic Accuracy of Point-of-Care Fluorescence Imaging for the Detection of Bacterial Burden in Wounds: Results from the 350-Patient Fluorescence Imaging Assessment and Guidance Trial

**DOI:** 10.1089/wound.2020.1272

**Published:** 2021-02-01

**Authors:** Lam Le, Marc Baer, Patrick Briggs, Neal Bullock, Windy Cole, Daniel DiMarco, Rachel Hamil, Khristina Harrell, Maria Kasper, Weili Li, Keyur Patel, Matthew Sabo, Kerry Thibodeaux, Thomas E. Serena

**Affiliations:** ^1^ The Heal Clinic, Tulsa, Oklahoma, USA.; ^2^ Foot & Ankle Center, Bryn Mawr, Pennsylvania, USA.; ^3^ HCA-Houston Healthcare Gulf Coast Foot and Ankle Specialists, Webster, Texas, USA.; ^4^ Royal Research Corp, Pembroke Pines, Florida, USA.; ^5^ Kent State University College of Podiatric Medicine, Kent, Ohio, USA.; ^6^ St. Vincent Wound & Hyperbaric Centre, Erie, Pennsylvania, USA.; ^7^ St. Mary's Center for Wound Healing, Athens, Georgia, USA.; ^8^ SerenaGroup Research Foundation, Cambridge, Massachusetts, USA.; ^9^ Martin Foot and Ankle, York, Pennsylvania, USA.; ^10^ Li & Li Statistical Consulting, Toronto, Canada.; ^11^ Armstrong County Memorial Hospital, Kittanning, Pennsylvania, USA.; ^12^ The Foot and Ankle Wellness Center of Western PA, Butler, Pennsylvania, USA.; ^13^ The Wound Treatment Center at Opelousas General Health System, Opelousas, Louisiana, USA.

**Keywords:** diagnostic accuracy, fluorescence imaging, wound assessment, wound infection

## Abstract

**Objective:** High bacterial load contributes to chronicity of wounds and is diagnosed based on assessment of clinical signs and symptoms (CSS) of infection, but these characteristics are poor predictors of bacterial burden. Point-of-care fluorescence imaging (FL) MolecuLight *i:X* can improve identification of wounds with high bacterial burden (>10^4^ colony-forming unit [CFU]/g). FL detects bacteria, whether planktonic or in biofilm, but does not distinguish between the two. In this study, diagnostic accuracy of FL was compared to CSS during routine wound assessment. Postassessment, clinicians were surveyed to assess impact of FL on treatment plan.

**Approach:** A prospective multicenter controlled study was conducted by 20 study clinicians from 14 outpatient advanced wound care centers across the United States. Wounds underwent assessment for CSS followed by FL. Biopsies were collected to confirm total bacterial load. Three hundred fifty patients completed the study (138 diabetic foot ulcers, 106 venous leg ulcers, 60 surgical sites, 22 pressure ulcers, and 24 others).

**Results:** Around 287/350 wounds (82%) had bacterial loads >10^4^ CFU/g, and CSS missed detection of 85% of these wounds. FL significantly increased detection of bacteria (>10^4^ CFU/g) by fourfold, and this was consistent across wound types (*p* < 0.001). Specificity of CSS+FL remained comparably high to CSS (*p* = 1.0). FL information modified treatment plans (69% of wounds), influenced wound bed preparation (85%), and improved overall patient care (90%) as reported by study clinicians.

**Innovation:** This novel noncontact, handheld FL device provides immediate, objective information on presence, location, and load of bacteria at point of care.

**Conclusion:** Use of FL facilitates adherence to clinical guidelines recommending prompt detection and removal of bacterial burden to reduce wound infection and facilitate healing.

**Figure:**
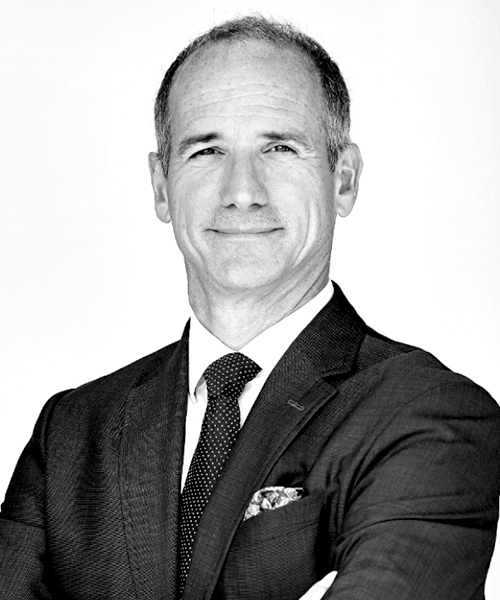
Thomas E. Serena, MD

## Introduction

An estimated 1–2% of the population in developed countries will experience a chronic wound in their lifetime^1^ and the incidence of wounds continues to rise as the population ages and comorbidities mount.^2^ As a result, management of chronic wounds accounts for >5% of total health care expenditures in the United States and United Kingdom.^3–6^

Chronic wounds fail to progress through a timely sequence of repair. It is known that increased microbial load is a key predictor of nonhealing wounds.^7,8^ Proliferation of bacteria resulting in moderate-to-heavy loads (>10^4^ colony-forming units [CFU]/g) delays healing^9–11^ and increases the risk of wound complications, including infection, sepsis, and amputation.^12–14^ Guidelines advise that early diagnosis of high bacterial burden is essential to prevent the wound from progression to local or systemic infection.^15^ To reduce bacterial burden, clinicians choose from an armamentarium of antiseptic wound cleansers, debridement techniques, and antimicrobial options. This is done without objective information on bacteria at point-of-care and without information on treatment efficacy.

## Clinical Problem Addressed

Treatment selection at point-of-care is largely based on evaluation of clinical signs and symptoms (CSS) of infection or high bacterial loads. However, numerous studies have reported that patients with high bacterial burden are frequently asymptomatic.^11,16,17^ Furthermore, comorbidities in wound patients (*e.g*., diabetes and autoimmune disease) can blunt immune responses and exacerbate patient-to-patient variability of CSS.^18^ Together, this results in poor sensitivity of CSS for detection of infection,^16,17,19^ hindering immediate identification of wounds with high bacterial burden. Quantitative tissue cultures of wound biopsies are the reference standard to quantify bacterial load, but prolonged turnaround time between biopsy and microbiological results limits the rapid decision making needed to effectively manage bacterial burden in wounds. The relative inconsistency of CSS and delays in results from microbiological culture and PCR analysis may explain why 12-week wound healing rates are below 60%^7^ and have remained stagnant over the past 40 years,^20^ despite tremendous advances in wound treatments.

**Figure 1.**
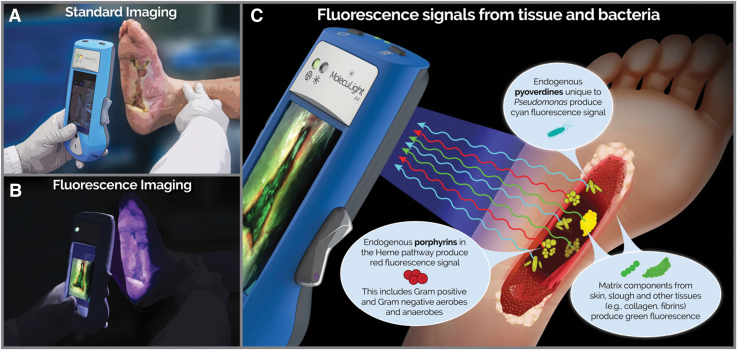
**(A)** Standard and **(B)** FL using the MolecuLight *i:X*. The *green* range finder LED indicates that the device is within optimal range (8–12 cm) and correctly positioned for imaging. Darkness is required (achieved by turning off room lights or using a DarkDrape) to capture fluorescence images. **(C)** When a wound is illuminated by the safe, *violet* (405 nm) light, components in the wound are excited up to a depth of 1.5 mm. Porphyrin-producing bacteria within the wound emit *red fluorescence* signals, *Pseudomonas aeruginosa* emits cyan fluorescence signals, and tissue components (*e.g*., collagen and fibrins) emit *green fluorescence* signals. An optical filter on the device captures these relevant signals and prevents reflected *violet* light from contaminating the image without any digital processing. FL, fluorescence imaging.

To address the pervasive problem of bacteria-related delayed healing and facilitate a more proactive approach to treatment planning, objective diagnostic information on bacterial burden in wounds is needed. Point-of-care diagnosis of bacterial burden in wounds is achieved using a handheld fluorescence imaging (FL) device (MolecuLight *i:X*; MolecuLight, Inc., Toronto, Canada) that detects endogenous fluorescence from bacteria (at loads >10^4^ CFU/g).^21^ Macroscopic imaging of bacteria is not possible as bacteria themselves are microscopic. However, when bacteria accumulate at high loads (>10^4^ CFU/g), the fluorophores they collectively emit are detectable through FL. Under safe violet light illumination, common wound pathogens, including bacteria from the *Staphylococcus*, *Proteus*, *Klebsiella*, and *Pseudomonas* generas,^22,23^ endogenously emit red or cyan fluorescent signatures.^23–26^ By detecting these fluorescent signals, FL provides immediate information on bacterial location, without use of contrast agents (Fig. 1). Multiple clinical studies have consistently reported positive predictive values (PPV) of these fluorescent signals averaging 95.6% (range 87.5–100%) to detect moderate-to-heavy loads of bacteria, confirmed by microbiological analysis.^21,27–29^ Recent evidence indicates that the FL procedure facilitates more appropriate treatment selection and timing of advanced therapies (*e.g*., grafts and skin substitutes)^30^ in chronic wounds and burns^27,28,31–35^; however these studies lacked rigor and statistical power. The Fluorescence imaging Assessment and Guidance (FLAAG) study, a large, multicenter prospective controlled clinical trial targeting wounds of various type and duration, was established to evaluate the following: (1) whether FL improves detection of wounds with high (>10^4^ CFU/g) bacterial loads and (2) how point-of-care information on bacterial presence and location impacts treatment planning.

## Materials and Methods

### Study population and design

This prospective, single-blind, multicenter cross-sectional study (clinicaltrials.gov No. NCT03540004) had two independent co-primary endpoints: (1) superiority in sensitivity of CSS and FL (CSS+FL) versus CSS alone, to identify wounds with moderate-to-heavy (>10^4^ CFU/g bacterial load); and (2) noninferiority of specificity of CSS+FL versus CSS alone with region of indifference of 10% to identify wounds with moderate-to-heavy bacterial load. These co-primary endpoints were independent of each other. A sample size of 160 patients, consisting of 100 positive cases (bacterial loads of >10^4^ CFU/g) to demonstrate superiority in sensitivity and 60 negative cases (bacterial loads of <10^4^ CFU/g) to demonstrate noninferiority of specificity, was chosen to achieve >80% power for both primary endpoints. The study included adult (>18 years) patients presenting with wounds: 138 diabetic foot ulcers (DFUs), 106 venous leg ulcers (VLUs), 22 pressure ulcers (PUs), 60 surgical sites (SS), and 24 others of unknown infection status (Supplementary Fig. S1). To ensure adequate representation of wound variety, a minimum of 20 participants were recruited with each wound type (*e.g*., DFU, VLU, PU, and SS). Due to the high prevalence of patients with bacterial loads >10^4^ CFU/g, rolling recruitment was performed until a sufficient number of microbiologically negative wounds (<10^4^ CFU/g) to achieve statistical power was met, at enrollment of 371 patients. An independent third party (Ironstone Product Development, Toronto, ON) was used to control for bias and ensure appropriate blinding. Patients were recruited from 14 U.S. outpatient advanced wound care centers by 20 clinicians (12 podiatrists, 4 surgeons, 1 emergency room physician, 1 wound care physician, and 2 nurse practitioners). Patients were excluded if they had been treated with an investigational drug within the last month, had recently (<30 days) had a wound biopsy, were not able to consent, had any contraindications to routine wound care and/or monitoring, or if their wounds could not be imaged due to anatomical location. Only one wound per patient was eligible for inclusion. Before beginning the study, clinicians were provided with on-site and online training on the use of device, image interpretation, good clinical practice, and trial procedures. Clinicians were required to pass (>80%) a color blindness and image interpretation test before enrolling participants. The study was conducted in accordance with Health Insurance Portability and Accountability Act guidelines, adhered to tenets of the International Conference on Harmonization E6 Good Clinical Practice (ICH GCP) and the Declaration of Helsinki, and received ethics approval by an external institutional review board (Veritas IRB, Montreal, Canada).

### Assessment of CSS of infection and FL

**Figure 2.**
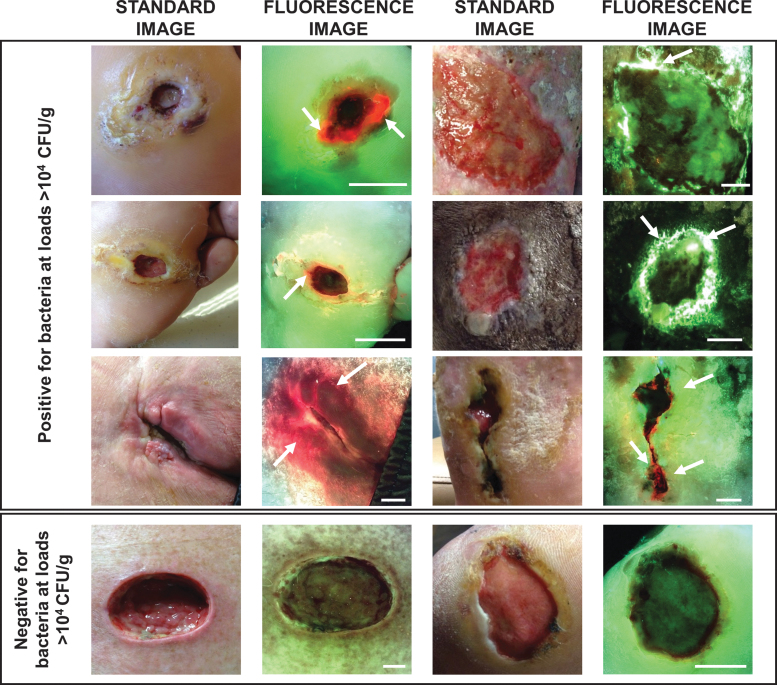
Representative fluorescence images of wounds that were positive or negative for moderate-to-heavy loads of bacteria (>10^4^ CFU/g) in and around the wound bed. *White arrows* indicate regions of *red* or *cyan fluorescence* from bacteria; scale bars represent 1 cm. CFU, colony-forming unit.

Clinicians reviewed patient history and visually inspected wounds for CSS using the International Wound Infection Institute (IWII) Wound Infection checklist.^15^ Assessment of infection was based on clinician judgment; wounds with ≥3 criteria present were considered positive for moderate-to-heavy (>10^4^ CFU/g) bacterial loads, per guidelines,^15^ but if one overwhelming sign or symptom was present, clinicians had the discretion to deem the wound positive for CSS. A 4-week treatment plan was created based on assessment of CSS. Immediately following CSS assessment, standard and fluorescence images were captured with the FL device. To ensure uniform FL, the device is held at a 90° angle to the wound. The device's LEDs emit safe 405 nm violet light to excite fluorophores in the wound up to a penetration depth of 1.5 mm.^36^ This excitation wavelength causes most bacterial species in wounds to emit a red fluorescent signal due to endogenous porphyrins in the heme pathway.^23,25^ While *Pseudomonas aeruginosa* also produces porphyrins,^37^ it uniquely produces a predominant cyan fluorescent signal due to endogenous pyoverdine, a virulence factor.^26^ These fluorescent signals from bacteria that accumulate in a region of the wound at loads >10^4^ CFU/g are detectable by the device.^21,29^ Specialized optical filters on the device allow transmission of only relevant fluorescence from tissue and bacteria.^36^ Connective tissues (*e.g*., collagen) produce green fluorescent signals^23,25,26,38^ and flaky skin appears a brighter green with white edges. Images where red or cyan fluorescence was observed by clinicians were considered positive for moderate-to-heavy bacterial loads (>10^4^ CFU/g)^21^ (Fig. 2). A new treatment plan was documented incorporating information about bacterial fluorescence. Clinicians then completed a survey indicating how FL influenced diagnosis of bacterial burden in the wound, guided procedure, and treatment selection (*i.e*., frequency of treatment, including cleaning, debridement, and use of topical antimicrobials and antibiotics), or influenced patient care.

### Microbiological analysis of total bacterial load

Punch biopsies from wounds were collected to quantify total bacterial load. Up to three biopsies (6 mm diameter) were obtained under local anesthetic: a biopsy from the wound center, or if applicable, a biopsy outside of the wound center from a region of the wound positive for bacterial fluorescence, or region positive for CSS. In wounds where bacterial fluorescence was observed, clinicians were directed to collect a biopsy from the region of the wound that was brightest for bacterial fluorescence. Biopsy samples were cut to a depth of 2 mm (to restrict bacterial contents to the penetration depth of imaging device) and transported in Remel ACT-II transport media to a central laboratory (Eurofins Central Laboratory, Lancaster, PA) for microbiological culture analysis of load and species. Fluorescence can only be detected from bacteria that are alive, thus necessitating the use of quantitative culture analysis to confirm the total bacterial loads detected by FL. This method may not fully capture the microbiological diversity in the wound, including some fastidious bacterial species; therefore, every effort was made to provide optimal conditions for bacteria that are challenging to culture. To prepare for analysis, a small portion of the tissue was prepared for Gram staining on a sterile slide. The remaining biopsy sample was homogenized and serially diluted^39^ for quantitative microbiological analysis (range of detection from 0 to 10^9^ CFU/g). Diluted biopsy homogenates were cultured on BAP/Chocolate agar (nonselective growth), Columbia CAN agar (select gram positive), MacConkey agar (selective gram negative), or Brucella agar (anaerobes) and incubated at 35°C in the appropriate atmosphere. Aerobe cultures were assessed for growth after 24 h of incubation and incubated up to 48 h; anaerobes were assessed after 48 h of incubation, and then reviewed every 24 h up to 7 days. A wound was considered microbiologically positive if the total bacterial load (the sum of all bacteria from any biopsy) was >10^4^ CFU/g. Matrix assisted laser desorption ionization-time of flight mass spectrometry (Bruker Daltonics) was used to identify bacterial species, as previously described.^40^ Microbiologists were blinded to the results of the CSS assessment and FL.

### Statistical analysis

One-sided exact McNemar tests were used for comparisons of sensitivity, specificity, and accuracy of detecting bacterial loads >10^4^ CFU/g. Comparisons of predictive values (PPV and negative predictive value [NPV]) were performed using an asymptotic method as described by Moskowitz and Pepe.^41^ Sample proportions and 95% confidence intervals (CIs) were used to estimate the diagnostic accuracy characteristics. Fisher's exact test was performed to assess association between fluorescence diagnosis (FL+ or FL−) and reported survey outcomes; statistical significance was set at *p* = 0.05. All analyses were performed using R version 3.6.2.

## Results

Between May 2018 and April 2019, 371 patients with various wound types (DFUs, VLUs, PUs, SS, and others) were screened. Of the 371 patients screened, only 4 (1.1%) were excluded from the study and microbiology data were completed for 350. Basic demographic information along with antibiotic use, wound type, wound duration, and total bacterial load are reported in Table 1. Mean (standard deviation [SD]) age of participants was 60.2 (12.4) and 35.7% were female. Wound duration exceeded 3 months in 69.7% of wounds and delayed healing was observed in 52.9%. No serious adverse event resulting from use of the device was reported.^42^

**Table 1.** Baseline characteristics of study participants

Characteristic	All Patients (*n* = 350)	Microbiology Positive (*n* = 287)	Microbiology Negative (*n* = 63)	*p*
	*N* (%)	*N* (%)	*N* (%)	
Age mean (SD)	60.19 (12.44)	59.95 (12.11)	61.27 (13.87)	0.45
Female	125.00 (35.71)	87 (30.31)	38 (60.32)	**<0.001**
Systemic antibiotic use (yes)	90 (25.71)	56 (19.51)	34 (53.97)	**<0.001**
Delayed healing present	185 (52.86)	158 (55.05)	27 (42.86)	**0.094**
Fitzpatrick score (skin tone)				
Light (I or II)	224 (64.00)	179 (62.37)	45 (71.43)	0.50
Medium (III or IV)	83 (23.71)	74 (25.78)	9 (14.29)	
Dark (V or VI)	43 (12.29)	34 (11.85)	9 (14.29)	
Wound type				
DFU	138 (39.43)	123 (42.86)	15 (23.81)	**0.009**
PU	22 (6.29)	20 (6.97)	2 (3.17)	
SS	60 (17.14)	44 (15.33)	16 (25.40)	
VLU	106 (30.29)	79 (27.53)	27 (42.86)	
Other	24 (6.86)	21 (7.32)	3 (4.76)	
Wound duration				
<3 Months	106 (30.29)	79 (27.53)	27 (42.86)	**0.008**
3–12 Months	118 (33.71)	93 (32.40)	25 (39.68)	
>12 Months	126 (36.00)	115 (40.07)	11 (17.46)	
Median (range) total bacterial load		1.80 × 10^6^ (0.00–7.70 × 10^9^)		

Wounds that were “microbiology positive” had bacterial loads >10^4^ CFU/g. Fischer's exact test was used to compare microbiology-positive and microbiology-negative subsets of each characteristic described. Statistical significance was set at *p* = 0.05; bold values indicate significance.

CFU, colony-forming unit; DFU, diabetic foot ulcer; PU, pressure ulcer; SD, standard deviation; VLU, venous leg ulcer.

**Figure 3.**
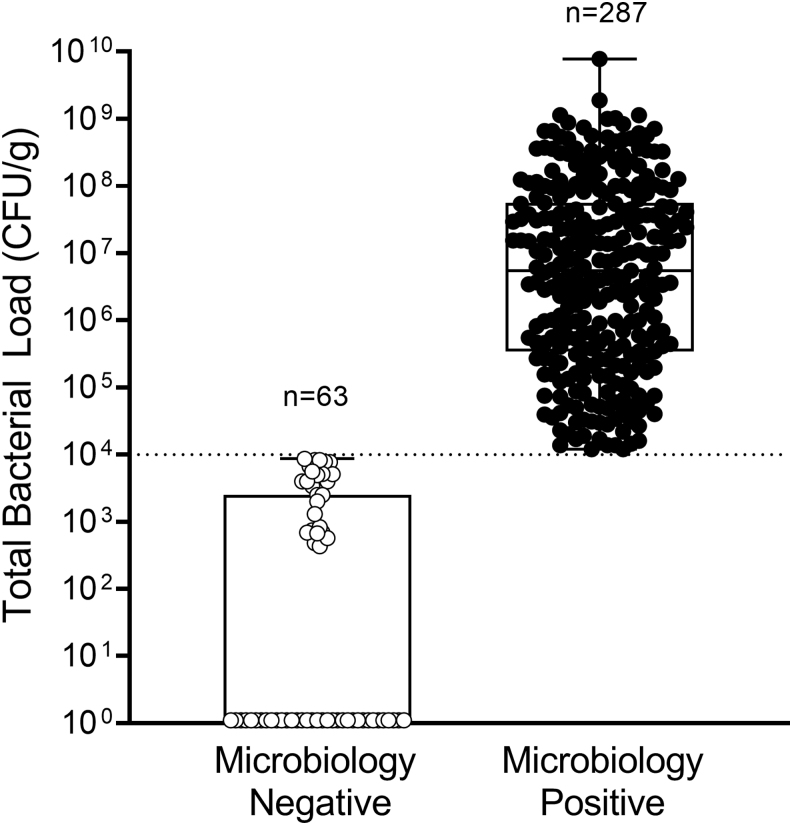
Box plot shows the distribution of total bacterial load (CFU/g) of each wound biopsied (*n* = 350 wounds total) based on whether wounds were microbiologically negative (bacterial load <10^4^ CFU/g; *n* = 63) or positive (>10^4^ CFU/g; *n* = 287). *Boxes* contain the 25th to 75th percentiles of data set, while *center line* indicates median bacterial load of all wounds (10^6^ CFU/g). *Black whiskers* represent minimum and maximum values. *Dashed line* indicates lowest threshold (10^4^ CFU/g) at which bacteria can be detected using FL. Of the microbiology-negative wound biopsies, 36 had total bacterial load of 0.

In 82% (287/350) of wounds, bacterial loads >10^4^ CFU/g were observed, confirmed by microbiological analysis (Fig. 3). Median (range) total bacterial load of all wounds was 1.8 × 10^6^ CFU/g (0.0–7.7 × 10^9^ CFU/g). A higher proportion of males (69.7%) than females (30.3%) had microbiology-positive wounds (>10^4^ CFU/g). Of the microbiology positive wounds, 19.5% were on systemic antibiotics, and bacterial load of these wounds averaged (SD) 1.4 × 10^7^ CFU/g (3.1 × 10^7^ CFU/g); over 50% of microbiology-negative wounds (<10^4^ CFU/g) were on systemic antibiotics. Bacterial loads >10^4^ CFU/g were most prevalent in DFUs and wounds of ≥12 month duration. Of the 350 wounds in the study, 183 (52.3%) had bacterial loads >10^6^ CFU/g, which some consider to be indicative of infection^17^; in 16.9% (59/350) of wounds, bacterial loads >10^8^ CFU/g were observed, while 18% (63/350) of wounds had bacterial loads <10^4^ CFU/g. One hundred and six different bacterial species (51 genera) were detected from 1,053 isolates; species detected included the following: 68 gram positive, 38 gram negative, 78 aerobes, and 28 anaerobes. In 85.7% (246/287) of microbiology-positive wounds (loads >10^4^ CFU/g), mixed bacterial colonization was present. *Staphylococcus aureus* was the most prevalent species observed, present in 71.1% of microbiology-positive wounds. *P. aeruginosa* was prevalent in 13.9% (40/287) of microbiology-positive wounds and was associated with presence of cyan fluorescence, as expected. Supplementary Table S1 lists bacterial species detected from all study wounds. An average of 2.8 bacterial species was detected per biopsy collected from the center of the wound. In most wounds, the center of the wound was also the brightest region of fluorescence. However, in 78 wounds, an additional FL-guided biopsy was collected outside the wound center. From these FL-guided biopsies taken outside of the wound center, an average of 3.1 bacterial species was detected. This was significantly higher than the average number of bacterial species detected in biopsies collected from the center of the same wound (2.2; *p* < 0.001). The inclusion of 98.9% (367/371) of the population screened suggests that these findings are representative of bacterial loads in typical wound populations.

**Figure 4.**
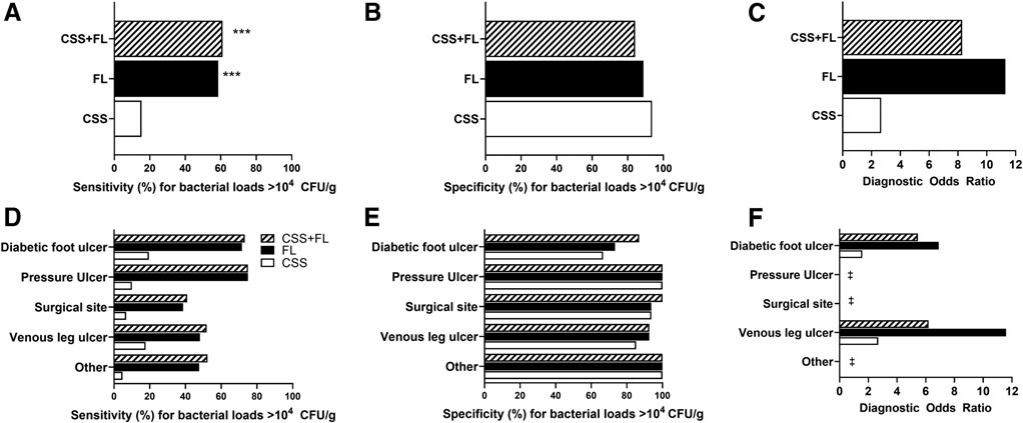
CSS of infection combined with FL were compared with CSS and FL alone at the participant level for sensitivity **(A)**, specificity **(B),** and DOR **(C)** (*n* = 350). Comparisons were also made between CSS, FL, and CSS+FL for each wound type **(D–F)**. ***p < 0.001 derived from a one-sided McNemar exact test. ^‡^When specificity was 100%, a DOR could not be calculated and compared between groups. CSS, clinical signs and symptoms; DOR, diagnostic odds ratio.

**Figure 5.**
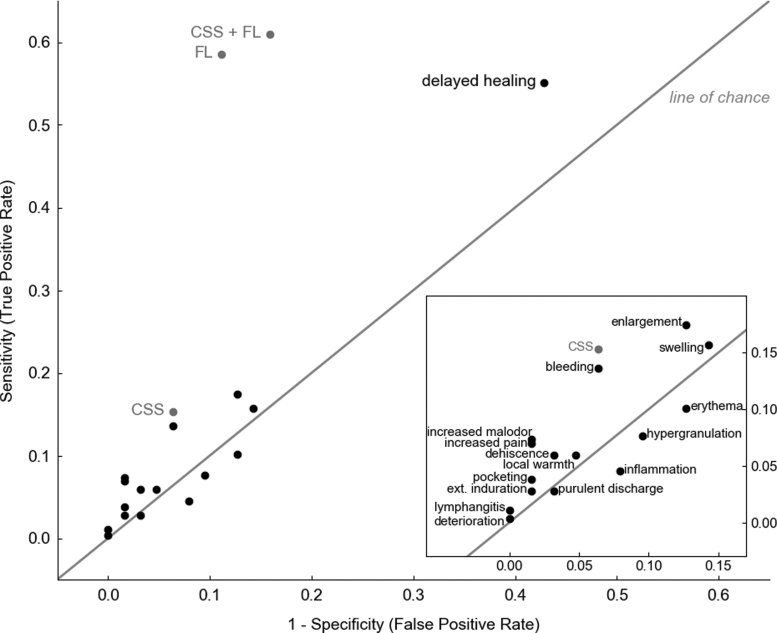
Scatter plot (pairs of sensitivity, 1-specificity) comparing discriminative power of CSS of infection (based on IWII criteria^14^), individual signs of infection, FL, and CSS+FL. Values in the *top left corner* indicate high discriminative power. Erythema, hypergranulation, inflammation, and purulent discharge all fell below the line of chance indicating they were no better than “flipping a coin” at predicting bacterial loads >10^4^ CFU/g in wounds. IWII, International Wound Infection Institute.

Diagnostic accuracy of FL was assessed on its own and in combination with information provided by CSS assessment (CSS+FL). Clinicians diagnosed 302/350 wounds as negative for CSS. Addition of FL to CSS improved sensitivity (61.0% [95% CI, 55.3–66.6%]) to detect wounds with bacterial loads >10^4^ CFU/g by fourfold compared to CSS alone (15.33% [95% CI, 11.16–19.50]; *p* < 0.001, Fig. 4A), consistent across wound types (Fig. 4D). Sensitivity of FL was comparable to CSS+FL. Detection of false positives using CSS and FL was rare, resulting in specificity of 84.1% (95% CI, 75.1–93.2%; Fig. 4B) of CSS+FL, which was comparable to CSS. Specificity of FL remained similarly high relative to CSS across all wound types (Fig. 4E). Diagnostic odds ratio of CSS+FL was 8.3 (95% CI, 4.1–17.0), and was 3.1-fold higher than CSS (2.7 [95% CI 0.9–7.7]; Fig. 4C). PPV of FL (either alone or in combination with CSS) was comparably high (96.0, 95% CI [93.1–98.9] and 94.6, 95% CI [91.3–97.9], respectively) to CSS alone (91.7, 95% CI [83.9–99.5]), but NPV and accuracy of CSS+FL were significantly increased by 64.4% and 2.2-fold, respectively, compared to CSS (Table 2; *p* < 0.001). CSS alone had poor discriminative power to predict wounds with high bacterial loads (Fig. 5); FL drove improvements in discriminative power to identify wounds with bacterial burden >10^4^ CFU/g at point of care. With FL, high bacterial burden was identified in 131 wounds otherwise missed by CSS. FL provided additional benefits at the time of diagnosis by locating bacterial burden outside of the wound bed in 128/302 (42.4%) wounds negative for CSS. The enhanced sensitivity, accuracy, and discriminative power of FL compared to CSS resulted in identification of a larger proportion of wounds with bacterial loads >10^4^ CFU/g.

**Table 2.** Estimates of positive predictive value, negative predictive value, and accuracy for detection of bacterial loads >10^4^ CFU/g

	CSS	CSS+FL	FL	CSS vs. CSS+FL	CSS vs. FL
	% (95% CI)			*p*	*p*
PPV	91.67 (83.85–99.49)	94.59 (91.34–97.85)	96.00 (93.10–98.90)	0.19	0.14
NPV	19.54 (15.06–24.01)	32.12 (25.00–39.25)	32.00 (25.09–38.91)	**<0.001**	**<0.001**
Accuracy	29.43 (24.90–34.41)	65.14 (60.01–69.95)	64.00 (58.84–68.85)	**<0.001**	**<0.001**

Values in bold indicate significance.

PPV, NPV, and accuracy were estimated for CSS, CSS+FL, and FL using microbiological analysis of total bacteria load to serve as ground truth. CSS of infection combined with FL was compared with CSS and FL alone at the participant level. All *p*-values were derived from one-sided tests.

CI, confidence interval; CSS, clinical signs and symptoms; FL, fluorescence imaging; NPV, negative predictive value; PPV, positive predictive value.

**Figure 6.**
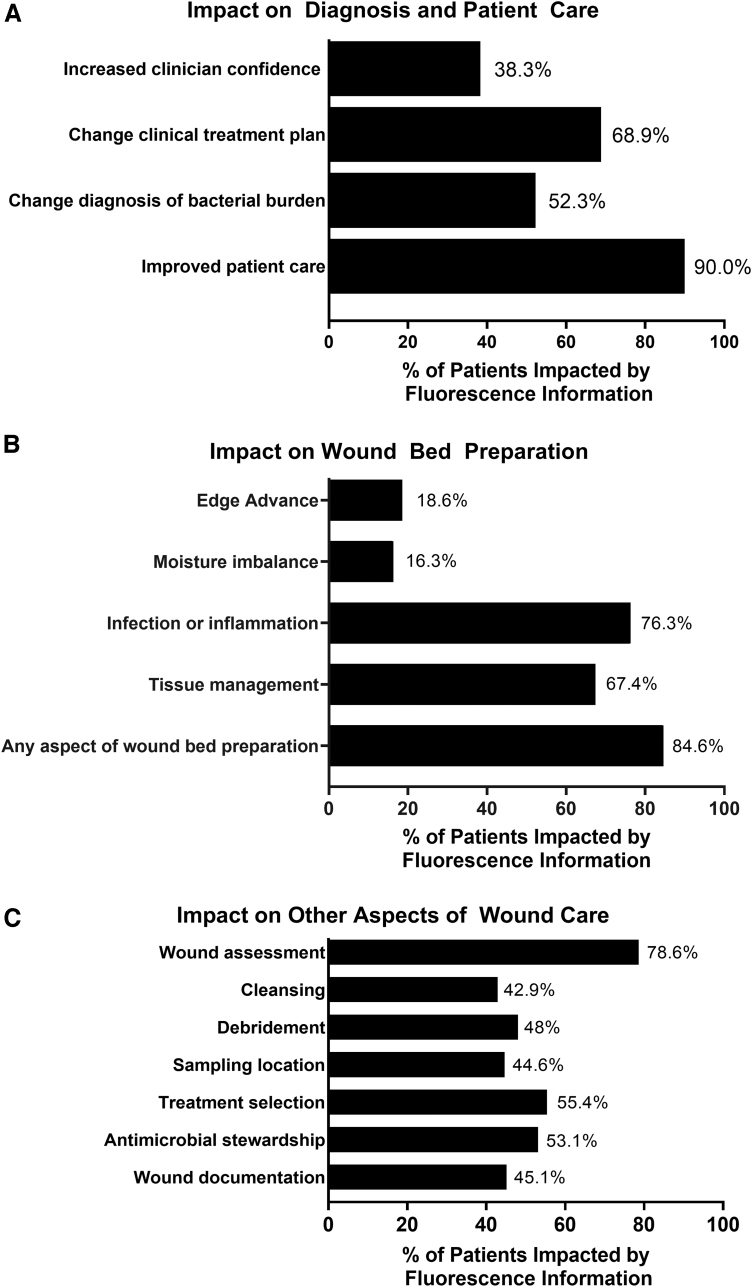
Impact of FL on care plan. Clinicians completed a survey on utility of fluorescence information after capturing images. Clinicians reported on how FL information impacted diagnosis and patient care **(A)**, wound bed preparation **(B)**, and other aspects of wound care **(C)**. Values indicate the percent of wounds impacted by FL information.

The impact of FL information on care planning was evaluated using a clinician survey. The survey asked clinicians to report which aspects of wound care were most impacted by FL. Clinicians reported that FL resulted in improvements to patient care (which includes wound bed preparation, treatment planning, patient engagement, and monitoring treatment efficacy) in 90.0% of study wounds. FL information also resulted in changes to diagnosis of bacterial burden in 52.3% of wounds (Fig. 6). The objective, diagnostic information provided by FL changed clinical treatment plans in 68.9% of wounds (Fig. 6A). FL information guided wound bed preparation in 84.6% of wounds; and had the greatest impact on primarily tissue management (67.4%) and infection control (76.3%; Fig. 6B). Wound care decision making stems from assessment; thus, not surprisingly, assessment was heavily influenced by FL-information (78.6%). Downstream aspects of care, including sampling location (44.6% of wounds), cleaning (42.9%), debridement (48.0%), treatment selection (55.4%), and wound documentation (45.1%), were also influenced (Fig. 6C). Table 3 summarizes the aspects of care that were impacted by fluorescence information and compares impact of that information in wounds deemed fluorescence (bacteria) positive versus fluorescence negative. As expected, changes to care plan, (with the exception of wound assessment, moisture imbalance, and edge advance), were more prevalent among wounds positive for bacterial fluorescence compared to those negative for bacterial fluorescence (*p* < 0.001), indicating that it was primarily the enhanced detection of bacteria provided by fluorescence information that significantly influenced clinicians' care planning.

**Table 3.** Impact of fluorescence imaging on care plan

	No./total (%)	FL+	FL−	*p*
Impact on diagnosis and patient care				
Improved patient care	315/350 (90.00)	169/315 (53.65)	146/315 (46.35)	**<0.001**
Changed diagnosis of bacterial burden	183/350 (52.29)	141/183 (77.05)	42/183 (22.95)	**<0.001**
Changed clinic treatment plan	241/350 (68.86)	148/241 (61.41)	93/241 (38.59)	**<0.001**
Increased clinician confidence (if no change to wound assessment)	134/350 (38.29)	41/134 (30.60)	93/134 (69.40)	**<0.001**
Aspects of wound bed preparation influenced by FL				
Any aspect of wound bed preparation	296/350 (84.57)	160/296 (54.05)	136/296 (45.95)	**<0.001**
Tissue management	236/350 (67.43)	131/236 (55.51)	105/236 (44.49)	**0.004**
Infection or inflammation	267/350 (76.29)	158/267 (59.18)	109/267 (40.82)	**<0.001**
Moisture imbalance	57/350 (16.29)	27/57 (47.37)	30/57 (52.63)	0.77
Edge advance	65/350 (18.57)	32/65 (49.23)	33/65 (50.77)	>0.99
Aspects of wound care influenced by FL				
Wound assessment	275/350 (78.57)	142/275 (51.64)	133/275 (48.36)	0.30
Cleansing	150/350 (42.86)	95/150 (63.33)	55/150 (36.67)	**<0.001**
Debridement	168/350 (48.00)	105/168 (62.50)	63/168 (37.50)	**<0.001**
Sampling location	156/350 (44.57)	121/350 (77.56)	35/156 (22.44)	**<0.001**
Treatment selection	194/350 (55.43)	116/194 (59.79)	78/194 (40.21)	**<0.001**
Antimicrobial stewardship	186/350 (53.14)	120/186 (64.52)	66/186 (35.48)	**<0.001**
Wound documentation	158/350 (45.14)	97/158 (61.39)	61/158 (38.61)	**<0.001**

Clinicians completed a survey on utility of fluorescence information after capturing images. The total number of participants where fluorescence information influenced care plan is listed in column 2. For each survey item, a Fischer's exact test was performed to assess differences between wounds deemed positive (FL+) or negative (FL−) for bacterial fluorescence. Statistical significance was set at *p* = 0.05; values in bold indicate significance.

## Discussion

Bacterial load in wounds is underestimated and the incidence of infection in the wound care population is underreported,^17,18^ and therefore undertreated. The presence and severity of bacterial loads in wounds are typically inferred from CSS.^43,44^ However, CSS is inherently subjective and frequently fails to detect wounds with moderate-to-heavy bacterial loads.^16,17^ More accurate methods to identify wounds with clinically significant loads of bacteria can facilitate better management of wounds according to standard of care practices.^15^ In this study, FL of bacteria to detect bacterial loads >10^4^ CFU/g was used in combination with standard of care assessment of CSS to determine if detection of wounds with high bacterial loads (>10^4^ CFU/g) could be improved. Microbiological analysis of wound biopsies revealed median bacterial load of 1.8 × 10^6^ CFU/g, with 36.6% of study wounds having bacterial loads >10^7^ CFU/g. At bacterial loads of 10^4^ CFU/g, clinical signs of infection may not manifest, but delayed wound healing is observed.^9,10^ CSS assessment failed to detect 84.7% (155/183) of wounds with bacterial loads >10^6^ CFU/g, a threshold that some consider indicative of infection.^18^ CSS (individual and combined criteria) had poor discriminatory power in identifying wounds with bacterial loads >10^4^ CFU/g. Delayed healing, which had high sensitivity, was the clear exception, but had poor specificity, likely due to presence of physical characteristics that may delay healing (*e.g*., presence of biofilm, vascular insufficiency, and poor offloading).^15,45^ Four signs of infection (purulent discharge, inflammation, hypergranulation, and erythema) fell below the line of chance and were ineffective at predicting bacterial loads >10^4^ CFU/g, consistent with previous reports.^16,17^ The poor discriminatory power of CSS would have resulted in 84.7% (243/287) of patients with bacterial loads >10^4^ CFU/g receiving inappropriate treatment to address bacteria at the time of assessment. Indeed, a recent meta-analysis of CSS effectiveness concludes “the apparent lack of utility of a combination of findings identified by infectious disease experts (Infectious Diseases Society of America criteria) as useful for diabetic foot infection is both surprising and disappointing, but highlights the difficulty in making the diagnosis.”^17^ To overcome stagnant wound healing trends, improved methods of identifying and treating bacterial load need to be prioritized.

Detection of bacteria in wounds using FL has been previously validated through *in vitro* and *in vivo* studies that elegantly demonstrated the correlation between intensity of fluorescent signal (from bacterial porphyrins) and bacterial load and showed that FL can detect both planktonic and biofilm-encased bacteria,^23,46^ although it cannot distinguish between these two states of bacteria. Biofilm detection and eradication are of tremendous importance in wound care, with biofilm prevalence estimated in up to 90% of chronic wounds.^47^ Even without distinguishing between planktonic and biofilm-encased bacteria, the ability of FL to detect bacteria in biofilm and target treatment to regions that potentially contain biofilm is a significant advancement for the field.

*In vitro* results lack the tissue in which wound bacteria are dispersed and other factors present in the wound that may influence capacity to detect high bacterial loads in wounds. This makes clinical studies critical to assess the true performance of this device to detect bacteria above 10^4^ CFU/g. Consistent with prior clinical studies,^33,35,48^ use of the FL diagnostic procedure to detect bacterial loads >10^4^ CFU/g resulted in higher sensitivity (4-fold) and accuracy (2.2-fold), enhanced detection of high bacterial burden in wounds otherwise missed by CSS, and immediately impacted treatment plans. Inaccurate or late diagnosis of bacteria and infection plagues chronic wounds at great costs to the patient and health care systems,^3,4,49^ and contributes to some of the 196 daily DFU-related amputations in the United States.^50^ Undertreatment and overtreatment can lead to suboptimal wound care, inflated costs, and antibiotic misuse.^51^ The robust performance characteristics of FL reported in this study demonstrate the applicability of this diagnostic procedure to facilitate earlier detection of detrimental wound bacterial burden.^15^

According to guidelines,^15^ intervention is mandated in wounds when bacterial colonization turns into local infection (≥10^6^ CFU/g). Intervention at this critical point prevents further escalation up the infection continuum and damage to host tissue. In this study, FL provided real-time evidence of high (>10^4^ CFU/g) bacterial loads in 131 wounds negative for CSS, prompting intervention in the form of bacterial-targeted therapies (*e.g*., cleansing, debridement, or use of antimicrobials). The inclusion of FL as part of routine wound assessment provided information on bacterial burden that led to additional improvements in care:

(1) Guided wound bed preparation in ≥90% of wounds in this and other studies.^35,52^ Information on location of bacterial burden at point of care has been shown to be highly impactful for debridement,^52,53^ selection of appropriate cleanser,^30^ and general wound bed preparation before application of advanced therapies.^30^ Advanced therapies such as cellular and tissue-based products and skin grafts often fail when high bacterial loads are present.^54–56^
(2) Alerted clinicians to unexpected location of bacterial loads.^27,52^ In this study, more than 80% of wounds (150/185) positive for fluorescence from bacteria had bacterial burden outside of the wound bed. Treatments to minimize bacterial load (*e.g*., debridement) are not typically targeted to this region^57^ and sampling is rarely performed outside of the wound bed.^58–60^ The FL information in this study provided objective evidence on location of bacteria to facilitate targeted eradication.
(3) Provided information on efficacy of antibiotics and guided stewardship decisions without delay.^35^ In this study, 56 microbiology-positive wounds were on systemic antibiotics at the time of enrollment. FL revealed the presence of red or cyan fluorescence, indicative of bacterial loads >10^4^ CFU/g in 39.3% (22/56) of these wounds. Biopsy analysis later confirmed the presence of bacteria at loads >10^4^ CFU/g in these wounds. Together, these findings suggest inadequacy of the antibiotic treatment that had been prescribed to those 22 patients.

A recent international position article on antimicrobial stewardship^51^ highlighted diagnostic uncertainty in wounds as a key factor contributing to antimicrobial misuse, and recommends the use of rapid, diagnostic testing to ensure judicious use of antimicrobials. In this study, we show evidence that supports this recommendation; FL resulted in more appropriate diagnosis of 46% of wounds with bacterial loads >10^4^ CFU/g compared to CSS and impacted antimicrobial stewardship decisions in 53.1% of wounds. Diagnostic imaging provides actionable information to better implement gold standard wound care.

### Strengths and limitations

This study of 350 patients included a heterogenous sample of wounds, across multiple clinical sites. The minimal participant exclusion criteria and diverse wound types included in the study increase the generalizability of results to the overall chronic wound population. Furthermore, the use of wound biopsy and culture analysis to confirm bacteria loads enhanced confidence in the diagnostic accuracy measures reported. However, there were limitations to these methodologies. First, due to the imprecision of soft tissue biopsy trimming, the biopsies were cut to a greater depth than the 1.5 mm excitation limit of the imaging device; thus, it is possible that the biopsy may have detected slightly more anaerobic bacteria than the device was able to. Second, the conditions of culture analysis are not favorable for fastidious bacteria and may have resulted in underreporting the diversity of bacteria species present in the wound. This study focused primarily on high bacterial loads as a contributor to delayed wound healing, but additional systemic factors that were not reported in this study, including vascular insufficiency^61^ and protease activity,^62^ must also be considered. Clinicians had limited experience using FL in a clinical context before the study, which may have contributed to lower sensitivity to detect bacteria at loads >10^4^ CFU/g than previously observed. In prior FL studies, sensitivity estimates ranging from 72% to 100% were reported, likely due to more clinician experience using the device.^21,28,29,63^ As with other diagnostic imaging modalities,^64–66^ we anticipate that the performance measures reported should be improved with increased experience.^67,68^ This single time point study meant that effectiveness of changes in treatment plan based on FL could not be measured. Longitudinal randomized controlled trials assessing wound healing may further elucidate the impact of point-of-care diagnostic imaging of bacteria. Evidence from small longitudinal observational studies demonstrate accelerated wound area reduction with use of FL.^32,53^ Due to the limited (1.5 mm) depth of excitation^36^ and inability to detect non-porphyrin-producing bacteria, including species from the *Streptococcus*, *Enterococcus*, and *Finegoldia* generas (which account for an estimated 12% of the most prevalent wound pathogens^23^ and rarely occur monomicrobially^69^), it is recommended that FL be used in combination with CSS.

## Conclusion

The severity of bacterial burden in wounds is grossly underappreciated. Our results from 350 wounds reveal failure of current standard-of-care assessment to detect 84.7% of wounds with bacterial loads >10^6^ CFU/g, which some suggest are indicative of infection.^18^ Incorporation of the noninvasive FL diagnostic procedure to wound assessment greatly improved detection of high bacterial burden across a variety of wound types and provided information on bacterial location at point of care. This represents a paradigm shift in wound assessment, in which clinicians now have immediate information on bacterial burden to guide treatment selection and inform the frequency of reassessment to determine the efficacy of selected treatments at point of care.^34,53^ The point-of-care information provided by FL facilitates a rapid switch to a more effective bacterial-targeting agent (*e.g*., cleanser and bandage).^34,70^ Study results, collected across 14 study sites from 20 clinicians of varying skill levels, indicate the widespread utility of FL to inform wound assessment, wound bed preparation, and overall treatment planning.

## Innovation

Despite advances in wound therapies, wound healing rates in the last 40 years have remained stagnant as clinicians continue to work blindly to address bacterial burden in wounds. In this study, FL increased detection of high loads (>10^4^ CFU/g) of bacteria by fourfold and informed the location and extent of bacteria in wounds. This actionable information enabled early detection of bacteria, especially in highly prevalent asymptomatic wounds, and allowed clinicians to treat bacterial burden without delays. Information provided by this noncontact point-of-care imaging device can be used to inform treatment planning and evaluate the efficacy of selected treatments.

**Key Findings**

- Eighty-two percent of study wounds (287/350) had clinically significant bacterial loads (>10^4^ CFU/g), which were missed by standard-of-care assessment of CSS of infection.
- Incorporation of MolecuLight *i:X* fluorescence imaging device with standard-of-care assessment of CSS increased point-of-care detection of wounds with high bacterial loads (>10^4^ CFU/g) by fourfold compared to CSS alone.
- Use of this noncontact point-of-care bacterial imaging device significantly impacted downstream aspects of patient care, including sampling location (44.6% of wounds), cleaning (42.9%) and debridement (48%), selection of antimicrobials (53.1%) and other treatments (55.4%).

## Supplementary Material

Supplemental data

Supplemental data

## About the Authors

**Lam Le, MD,** is medical director of the St. John Wound Center and a certified wound specialist. **Marc Baer, DPM,** practices at the Bryn Mawr Hospital Wound Care Center. **Patrick Briggs, DPM,** is currently part of Texas Gulf Coast Medical Group. **Neal Bullock, DPM,** is a podiatric surgery specialist practicing in Pembroke Pines, Florida. **Windy Cole, DPM,** is Director of Wound Care Research at Kent State University College of Podiatric Medicine. **Daniel DiMarco, DO,** is a family medicine specialist in Fairview, Pennsylvania. **Rachel Hamil, MD,** is a wound care and emergency medicine specialist in Athens, GA. **Khristina Harrell, RN,** is Chief Nursing Officer of the SerenaGroup research foundation. **Maria Kasper, DPM, FACFAS,** is the medical director at Martin Foot and Ankle, and is affiliated with Wellspan/York Hospital and UPMC Pinnacle Memorial Hospital. **Weili Li, PhD,** is cofounder of Li & Li Statistical Consulting. She holds a PhD in Biostatistics. **Keyur Patel, DO,** currently serves as Medical Co-Director of the Wound & Hyperbaric Center and attending Emergency Physician at Armstrong County Memorial Hospital. **Matthew Sabo, DPM, FACFAS,** is a Clinical Associate Professor at Temple University School of Podiatric Medicine and is the associate director of research at The Snyder Institute of Armstrong County Memorial Hospital. **Kerry Thibodeaux, MD, FACS, CWSP,** is a general surgeon in Opelousas, LA. **Thomas E. Serena, MD,** is Founder and Medical director of the SerenaGroup, a family of wound, hyperbaric, and research companies.

## Abbreviations and Acronyms

CFU: colony-forming units
CI: confidence interval
CSS: clinical signs and symptoms
DFU: diabetic foot ulcer
DOR: diagnostic odds ratio
FL: fluorescence imaging
NPV: negative predictive value
PPV: positive predictive value
PU: pressure ulcer
SD: standard deviation
SS: surgical site
VLU: venous leg ulcer

## References

[1] Gottrup F. A specialized wound-healing center concept: importance of a multidisciplinary department structure and surgical treatment facilities in the treatment of chronic wounds. Am J Surg 2004;187:38S–43S 15147991 10.1016/S0002-9610(03)00303-9

[2] Sen CK, Gordillo GM, Roy S, et al. Human skin wounds: a major and snowballing threat to public health and the economy. Wound Repair Regen 2009;17:763–771 19903300 10.1111/j.1524-475X.2009.00543.x PMC2810192

[3] Nussbaum SR, Carter MJ, Fife CE, et al. An economic evaluation of the impact, cost, and medicare policy implications of chronic nonhealing wounds. Value Health 2018;21:27–32 29304937 10.1016/j.jval.2017.07.007

[4] Guest JF, Ayoub N, McIlwraith T, et al. Health economic burden that different wound types impose on the UK's National Health Service. Int Wound J 2017;14:322–330 27229943 10.1111/iwj.12603 PMC7950097

[5] Phillips CJ, Humphreys I, Fletcher J, Harding K, Chamberlain G, Macey S Estimating the costs associated with the management of patients with chronic wounds using linked routine data. Int Wound J 2016;13:1193–1197 25818405 10.1111/iwj.12443 PMC7949824

[6] Brem H, Stojadinovic O, Diegelmann RF, et al. Molecular markers in patients with chronic wounds to guide surgical debridement. Mol Med 2007;13:30–39 17515955 10.2119/2006-00054.Brem PMC1869625

[7] Cho S. Development of a model to predict healing of chronic wounds within 12 weeks. Adv Wound Care 2020 [Epub ahead of print]; DOI: 10.1089/wound.2019.1091 PMC7522633 32941121

[8] Tuttle MS. Association between microbial bioburden and healing outcomes in venous leg ulcers: a review of the evidence. Adv Wound Care (New Rochelle) 2015;4:1–11 25566410 10.1089/wound.2014.0535 PMC4281836

[9] Caldwell MD. Bacteria and antibiotics in wound healing. Surg Clin North Am 2020;100:757–776 32681875 10.1016/j.suc.2020.05.007

[10] Xu L, McLennan SV, Lo L, et al. Bacterial load predicts healing rate in neuropathic diabetic foot ulcers. Diabetes Care 2007;30:378–380 17259515 10.2337/dc06-1383

[11] Browne AC, Vearncombe M, Sibbald RG High bacterial load in asymptomatic diabetic patients with neurotrophic ulcers retards wound healing after application of Dermagraft. Ostomy Wound Manage 2001;47:44–49 11890078

[12] Turtiainen J, Hakala T, Hakkarainen T, Karhukorpi J The impact of surgical wound bacterial colonization on the incidence of surgical site infection after lower limb vascular surgery: a prospective observational study. Eur J Vasc Endovasc Surg 2014;47:411–417 24512892 10.1016/j.ejvs.2013.12.025

[13] Gardner SE, Frantz RA Wound bioburden and infection-related complications in diabetic foot ulcers. Biol Res Nurs 2008;10:44–53 18647759 10.1177/1099800408319056 PMC3777233

[14] Misic AM, Gardner SE, Grice EA The wound microbiome: modern approaches to examining the role of microorganisms in impaired chronic wound healing. Adv Wound Care (New Rochelle) 2014;3:502–510 25032070 10.1089/wound.2012.0397 PMC4086514

[15] International Wound Infection Institute (IWII). Wound infection in clinical practice. London, UK: Wounds International, 2016

[16] Gardner SE, Frantz RA, Doebbeling BN The validity of the clinical signs and symptoms used to identify localized chronic wound infection. Wound Repair Regen 2001;9:178–186 11472613 10.1046/j.1524-475x.2001.00178.x

[17] Reddy M, Gill SS, Wu W, Kalkar SR, Rochon PA Does this patient have an infection of a chronic wound? JAMA 2012;307:605–611 22318282 10.1001/jama.2012.98

[18] Gardner SE, Hillis SL, Frantz RA Clinical signs of infection in diabetic foot ulcers with high microbial load. Biol Res Nurs 2009;11:119–128 19147524 10.1177/1099800408326169 PMC2752486

[19] Serena TE, Hanft JR, Snyder R The lack of reliability of clinical examination in the diagnosis of wound infection: preliminary communication. Int J Low Extrem Wounds 2008;7:32–35 18372267 10.1177/1534734607313984

[20] Fife CE, Eckert KA, Carter MJ Publicly reported wound healing rates: the fantasy and the reality. Adv Wound Care (New Rochelle) 2018;7:77–94 29644145 10.1089/wound.2017.0743 PMC5833884

[21] Rennie MY, Lindvere-Teene L, Tapang K, Linden R Point-of-care fluorescence imaging predicts the presence of pathogenic bacteria in wounds: a clinical study. J Wound Care 2017;26:452–460 28795890 10.12968/jowc.2017.26.8.452

[22] Cavallaro G, Decaria L, Rosato A Genome-based analysis of heme biosynthesis and uptake in prokaryotic systems. J Proteome Res 2008;7:4946–4954 18808173 10.1021/pr8004309

[23] Jones LM, Dunham D, Rennie MY, et al. In vitro detection of porphyrin-producing wound bacteria with real-time fluorescence imaging. Future Microbiol 2020;15:319–332 32101035 10.2217/fmb-2019-0279

[24] Nitzan Y, Kauffman M Endogenous porphyrin production in bacteria by δ-aminolaevulinic acid and subsequent bacterial photoeradication. Lasers Med Sci 1999;14:8

[25] Philipp-Dormston WK, Doss M Comparison of porphyrin and heme biosynthesis in various heterotrophic bacteria. Enzyme 1973;16:57–64 4208581 10.1159/000459362

[26] Meyer JM, Abdallah MA The fluorescent pigment of *Pseudomonas fluorescens*: biosynthesis, purification and physicochemical properties. Microbiology 1978;107:9

[27] Farhan N, Jeffery S Utility of MolecuLight i:X for managing bacterial burden in pediatric burns. J Burn Care Res 2020;41:328–338 31541236 10.1093/jbcr/irz167

[28] Hurley CM, McClusky P, Sugrue RM, Clover JA, Kelly JE Efficacy of a bacterial fluorescence imaging device in an outpatient wound care clinic: a pilot study. J Wound Care 2019;28:438–443 31295094 10.12968/jowc.2019.28.7.438

[29] Serena TE. Evaluation of MolecuLight i:X as an adjunctive fluorescence imaging tool to clinical signs and symptoms for the identification of bacteria-containing wounds. clinicaltrials.gov No. NCT035400042019

[30] Aung B. Can fluorescence imaging predict the success of CTPs for wound closure and save costs? Todays Wound Clinic 2019;13:22–25

[31] Blumenthal E, Jeffery SLA The use of the MolecuLight i:X in managing burns: a pilot study. J Burn Care Res 2018;39:154–161 28448296 10.1097/BCR.0000000000000565

[32] DaCosta RS, Kulbatski I, Lindvere-Teene L, et al. Point-of-care autofluorescence imaging for real-time sampling and treatment guidance of bioburden in chronic wounds: first-in-human results. PLoS One 2015;10:e0116623 25790480 10.1371/journal.pone.0116623 PMC4366392

[33] Hill R, Rennie MY, Douglas J Using bacterial fluorescence imaging and antimicrobial stewardship to guide wound management practices: a case series. Ostomy Wound Manage 2018;64:18–28 30212361

[34] Raizman R. Fluorescence imaging guided dressing change frequency during negative pressure wound therapy: a case series. J Wound Care 2019;28(Suppl 9):S28–S37 31509488 10.12968/jowc.2019.28.Sup9.S28

[35] Serena TE, Harrell K, Serena L, Yaakov RA Real-time bacterial fluorescence imaging accurately identifies wounds with moderate-to-heavy bacterial burden. J Wound Care 2019;28:346–357 31166857 10.12968/jowc.2019.28.6.346

[36] Rennie MY, Dunham D, Lindvere-Teene L, Raizman R, Hill R, Linden R Understanding real-time fluorescence signals from bacteria and wound tissues observed with the MolecuLight i:X(TM). Diagnostics (Basel) 2019;9:22 10.3390/diagnostics9010022 PMC6468690 30813551

[37] Amin RM, Bhayana B, Hamblin MR, Dai T Antimicrobial blue light inactivation of *Pseudomonas aeruginosa* by photo-excitation of endogenous porphyrins: in vitro and in vivo studies. Lasers Surg Med 2016;48:562–568 26891084 10.1002/lsm.22474 PMC4914480

[38] Zhao HL, Zhang CP, Zhu H, Jiang YF, Fu XB Autofluorescence of collagen fibres in scar. Skin Res Technol 2017;23:588–592 28513064 10.1111/srt.12375

[39] Buchanan K, Heimbach DM, Minshew BH, Coyle MB Comparison of quantitative and semiquantitative culture techniques for burn biopsy. J Clin Microbiol 1986;23:258–261 3084547 10.1128/jcm.23.2.258-261.1986 PMC268623

[40] Sauget M, Valot B, Bertrand X, Hocquet D Can MALDI-TOF mass spectrometry reasonably type bacteria? Trends Microbiol 2017;25:447–455 28094091 10.1016/j.tim.2016.12.006

[41] Moskowitz CS, Pepe MS Comparing the predictive values of diagnostic tests: sample size and analysis for paired study designs. Clin Trials 2006;3:272–279 16895044 10.1191/1740774506cn147oa

[42] Serena TE, Cole W, Coe S, et al. The safety of punch biopsies on hard-to-heal wounds: a large multicentre clinical trial. J Wound Care 2020;29:S4–S7

[43] Bowler PG. Wound pathophysiology, infection and therapeutic options. Ann Med 2002;34:419–427 12523497 10.1080/078538902321012360

[44] Cutting KF, White RJ Criteria for identifying wound infection—revisited. Ostomy Wound Manage 2005;51:28–34 15695833

[45] Costerton JW, Stewart PS, Greenberg EP Bacterial biofilms: a common cause of persistent infections. Science 1999;284:1318–1322 10334980 10.1126/science.284.5418.1318

[46] Lopez AJ. In vivo detection of bacteria within a biofilm using a point-of-care fluorescence imaging device (Abstract). Virtual meeting: Symposium on Advanced Wound Care, 2020

[47] Attinger C, Wolcott R Clinically addressing biofilm in chronic wounds. Adv Wound Care (New Rochelle) 2012;1:127–132 24527292 10.1089/wound.2011.0333 PMC3839004

[48] Blackshaw EL, Jeffery SLA Efficacy of an imaging device at identifying the presence of bacteria in wounds at a plastic surgery outpatients clinic. J Wound Care 2018;27:20–26 29333929 10.12968/jowc.2018.27.1.20

[49] Olsson M, Jarbrink K, Divakar U, et al. The humanistic and economic burden of chronic wounds: a systematic review. Wound Repair Regen 2019;27:114–125 30362646 10.1111/wrr.12683

[50] Fakorede FA. Increasing awareness about peripheral artery disease can save limbs and lives. Am J Manag Care 2018;24(14 Spec No.):SP609 30620546

[51] Lipsky BA, Dryden M, Gottrup F, Nathwani D, Seaton RA, Stryja J Antimicrobial stewardship in wound care: a position paper from the British Society for Antimicrobial Chemotherapy and European Wound Management Association. J Antimicrob Chemother 2016;71:3026–3035 27494918 10.1093/jac/dkw287

[52] Raizman R, Dunham D, Lindvere-Teene L, et al. Use of a bacterial fluorescence imaging device: wound measurement, bacterial detection and targeted debridement. J Wound Care 2019;28:824–834 31825778 10.12968/jowc.2019.28.12.824

[53] Cole W, Coe S Use of a bacterial fluorescence imaging system to target wound debridement and accelerate healing: a pilot study. J Wound Care 2020;29(Suppl 7):S44–S52 10.12968/jowc.2020.29.Sup7.S44 32654620

[54] Hogsberg T, Bjarnsholt T, Thomsen JS, Kirketerp-Moller K Success rate of split-thickness skin grafting of chronic venous leg ulcers depends on the presence of *Pseudomonas aeruginosa*: a retrospective study. PLoS One 2011;6:e20492 21655269 10.1371/journal.pone.0020492 PMC3105064

[55] Xu Z, Hsia HC The impact of microbial communities on wound healing: a review. Ann Plast Surg 2018;81:113–123 29746280 10.1097/SAP.0000000000001450

[56] Zekri A KW. Success of skin grafting on a contaminated recipient surface. Eur J Plast Surg 1995;18:40–42

[57] Moelleken M, Jockenhöfer F, Benson S, Dissemond J Prospective clinical study on the efficacy of bacterial removal with mechanical debridement in and around chronic leg ulcers assessed with fluorescence imaging. Int Wound J 2020;17:101–1018 10.1111/iwj.13345 PMC7948916 32289211

[58] Copeland-Halperin LR, Kaminsky AJ, Bluefeld N, Miraliakbari R Sample procurement for cultures of infected wounds: a systematic review. J Wound Care 2016;25:S4–S6, S8–S10. 10.12968/jowc.2016.25.Sup4.S4 27068349

[59] Huang Y, Cao Y, Zou M, et al. A comparison of tissue versus swab culturing of infected diabetic foot wounds. Int J Endocrinol 2016;2016:8198714 27123004 10.1155/2016/8198714 PMC4829715

[60] Tedeschi S, Negosanti L, Sgarzani R, et al. Superficial swab versus deep-tissue biopsy for the microbiological diagnosis of local infection in advanced-stage pressure ulcers of spinal-cord-injured patients: a prospective study. Clin Microbiol Infect 2017;23:943–947 28433727 10.1016/j.cmi.2017.04.015

[61] Thomas HC. Checklist for factors affecting wound healing. Adv Skin Wound Care 2011;24:192 21422844 10.1097/01.ASW.0000396300.04173.ec

[62] McCarty SM, Percival SL Proteases and delayed wound healing. Adv Wound Care (New Rochelle) 2013;2:438–447 24688830 10.1089/wound.2012.0370 PMC3842891

[63] Jeffery S. The utility of MolecuLight bacterial sensing in the management of burns and traumatic wounds. Proceedings of SPIE 10863, Photonic Diagnosis and Treatment of Infections and Inflammatory Diseases II San Francisco, CL, 2019

[64] Cooper L, Gale A, Darker I, Toms A, Saada J. Radiology image perception and observer performance: How does expertise and clinical information alter interpretation? Stroke detection explored through eye-tracking. Proceedings of SPIE 7263, Medical Imaging 2009: Image Perception, Observer Performance, and Technology Assessment Lake Buena Vista, FL, 2009

[65] Wood G, Knapp KM, Rock B, Cousens C, Roobottom C, Wilson MR Visual expertise in detecting and diagnosing skeletal fractures. Skeletal Radiol 2013;42:165–172 22940835 10.1007/s00256-012-1503-5

[66] Nakashima R, Kobayashi K, Maeda E, Yoshikawa T, Yokosawa K Visual search of experts in medical image reading: the effect of training, target prevalence, and expert knowledge. Front Psychol 2013;4:166 23576997 10.3389/fpsyg.2013.00166 PMC3617447

[67] Esserman L, Cowley H, Eberle C, et al. Improving the accuracy of mammography: volume and outcome relationships. J Natl Cancer Inst 2002;94:369–375 11880475 10.1093/jnci/94.5.369

[68] Brealey S, Scally A, Hahn S, Thomas N, Godfrey C, Coomarasamy A Accuracy of radiographer plain radiograph reporting in clinical practice: a meta-analysis. Clin Radiol 2005;60:232–241 15664578 10.1016/j.crad.2004.07.012

[69] Wolcott RD, Hanson JD, Rees EJ, et al. Analysis of the chronic wound microbiota of 2,963 patients by 16S rDNA pyrosequencing. Wound Repair Regen 2016;24:163–174 26463872 10.1111/wrr.12370

[70] Hill R. How effective is your wound cleanser?: An evaluation using bacterial fluorescence imaging. San Antonio, Texas: SAWC Spring 2019, 2019

