# Supplementary figures and images for: Diagnostic Accuracy of Point-of-Care Fluorescence Imaging for the Detection of Bacterial Burden in Wounds: Results from the 350-Patient Fluorescence Imaging Assessment and Guidance Trial
Source: Adv Wound Care (New Rochelle). 2021 Feb 1;10(3):123–36. doi: 10.1089/wound.2020.1272 (PMC7876364; doi:10.1089/wound.2020.1272)

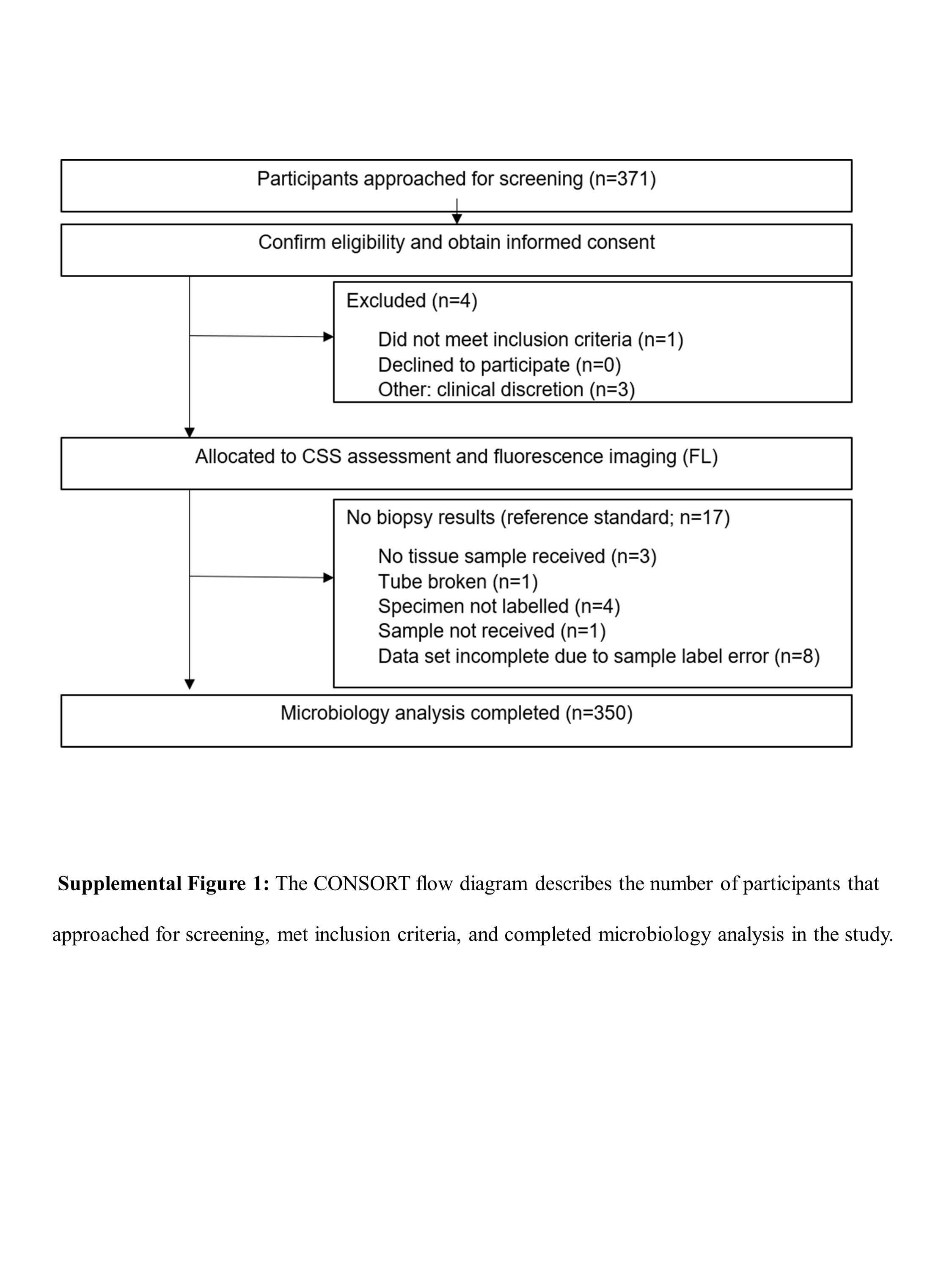

Supplement: Supplemental data [file Supp_Fig1.tif]
