# Supplementary material for: Diagnostic Accuracy of Point-of-Care Fluorescence Imaging for the Detection of Bacterial Burden in Wounds: Results from the 350-Patient Fluorescence Imaging Assessment and Guidance Trial
Source: Adv Wound Care (New Rochelle). 2021 Feb 1;10(3):123–36. doi: 10.1089/wound.2020.1272 (PMC7876364; doi:10.1089/wound.2020.1272)
Supplement: Supplemental data [file Supp_Table1.docx]

**Supplemental Table 1**

| **Genus** | **Species** | **Genus** | **Species** |
| --- | --- | --- | --- |
| Achromobacter | Achromobacter xylosoxidans | *Peptoniphilus* | Peptoniphilus asaccharolyticus |
| Acinetobacter | Acinetobacter baumannii |  | Peptoniphilus species |
|  | Acinetobacter calcoacetius | *Peptostreptococcus* | Peptostreptococcus anaerobius |
| Actinomyces | Actinomyces neuii |  | Peptostreptococcus prevotii |
|  | Actinomyces species | *Porphyromonas* | Porphyromonas asaccharolytica |
| Actinotignum | Actinotignum schaalii |  | Porphyromonas gingivalis |
|  | Actinotignum species |  | Porphyromonas species |
| Aerococcus | Aerococcus species | *Prevotella* | Prevotella bivia |
|  | Aerococcus viridans |  | Prevotella disiens |
| Alcaligenes | Alcaligenes faecalis |  | Prevotella species |
| Alloscardovia | Alloscardovia omnicolens | *Propionibacterium* | Propionibacterium acnes |
| Anaerococcus | Anaerococcus species | *Proteus* | Proteus hauseri |
|  | Anaerococcus tetradius |  | Proteus mirabilis |
| Arcanobacterium | Arcanobacterium haemolyticum |  | Proteus vulgaris |
| Arthrobacter | Arthrobacter species | *Providencia* | Providencia rettgeri |
| Bacteroides | Bacteroides fragilis |  | Providencia stuartii |
|  | Bacteroides fragilis group | *Pseudomonas* | Pseudomonas aeruginosa |
|  | Bacteroides thetaiotaomicron |  | Pseudomonas mendocina |
| Bordetella | Bordetella species | *Raoultella* | Raoultella ornithinolytica |
| Brevibacteruim | Brevibacterium casei | *Rothia* | Rothia mucilaginosa |
|  | Brevibacterium species |  | Rothia species |
| Campylobacter | Campylobacter ureolyticus | *Serratia* | Serratia marcescens |
| Citrobacter | Citrobacter freundii complex | *Staphylococcus* | Staphylococcus aureus |
|  | Citrobacter koseri |  | Staphylococcus capitis |
| Clostridium | Clostridium perfringens |  | Staphylococcus caprae |
|  | Clostridium ramosum |  | Staphylococcus cohnii |
| Corneybacterium | Corynebacterium jeikeium |  | Staphylococcus epidermidis |
|  | Corynebacterium species |  | Staphylococcus haemolyticus |
|  | Corynebacterium striatum |  | Staphylococcus hominis |
| Dermabacter | Dermabacter hominis |  | Staphylococcus lugdunensis |
| Enterobacter | Enterobacter aerogenes |  | Staphylococcus pettenkoferi |
|  | Enterobacter cloacae complex |  | Staphylococcus pseudintermedius |
| Enterococcus | Enterococcus avium |  | Staphylococcus schleiferi |
|  | Enterococcus casseliflavus |  | Staphylococcus simulans |
|  | Enterococcus faecalis |  | Staphylococcus warneri |
|  | Enterococcus faecium | *Stenotrophomonas* | Stenotrophomonas maltophilia |
|  | Enterococcus gallinarum | *Streptococcus* | Streptococcus agalactiae |
| Escherichia | Escherichia coli |  | Streptococcus anginosus |
| Eubacterium | Eubacterium aerofaciens |  | Streptococcus canis |
| Finegoldia | Finegoldia magna |  | Streptococcus constellatus |
| Fusobacterium | Fusobacterium species |  | Streptococcus dysgalactiae |
|  | Fusobacterium varium |  | Streptococcus gallolyticus |
| Gemella | Gemella morbillorum |  | Streptococcus Group C |
| Globicatella | Globicatella sanguinis |  | Streptococcus Group G |
|  | Globicatella species |  | Streptococcus intermedius |
| Helcococcus | Helcococcus kunzii |  | Streptococcus mitis |
| Klebsiella | Klebsiella oxytoca |  | Streptococcus mitis group |
|  | Klebsiella pneumoniae |  | Streptococcus oralis |
| Kocuria | Kocuria species |  | Streptococcus pyogenes |
| Lactobacillus | Lactobacillus species | *Trueperella* | Trueperella bernardiae |
| Leclercia | Leclercia adecarboxylata | *Vagococcus* | Vagococcus species |
| Morganella | Morganella morganii |  |  |
| Pantoea | Pantoea agglomerans | **Yeast Species** | |
| Parvimonas | Parvimonas micra | *Candida* | Parapsilosis |
| Pasteurella | Pasteurella multocida |  | Albicans |

**Supplemental Table 1. Bacterial and yeast species detected in biopsy samples from all study wounds.** The table lists the 106 species and 51 genera identified from the 350 wounds included in the study.
